# Neovascular Glaucoma Complicating Central Retinal Artery Occlusion Despite Cilioretinal Artery Sparing

**DOI:** 10.7759/cureus.43303

**Published:** 2023-08-10

**Authors:** Nur Aisyah Zakaria, Teck Chee Cheng, Rona A Nasaruddin, Jemaima Che Hamzah

**Affiliations:** 1 Department of Ophthalmology, Faculty of Medicine, Universiti Kebangsaan Malaysia, Kuala Lumpur, MYS; 2 Department of Ophthalmology, Hospital Canselor Tuanku Muhriz, Jalan Yaacob Latif, Kuala Lumpur, MYS

**Keywords:** carotid artery stenosis, central retinal artery occlusion (crao), carotid endarterectomy (cea), oct macula, visual prognosis, cilioretinal artery sparing

## Abstract

This case report aims to describe a case of unilateral central retinal artery occlusion (CRAO) with cilioretinal artery sparing, which was complicated by neovascular glaucoma (NVG). A 75-year-old Indian woman with underlying normal tension glaucoma presented with the sudden onset of painless generalized blurring of the right eye's vision for a week. Her right eye vision was hand motion with the presence of a right relative afferent pupillary defect. Fundus examination revealed retinal whitening over the macula sparing the papillomacular bundle with generalized retinal arteriolar attenuation, which was suggestive of right CRAO with cilioretinal artery sparing. Systemic examination revealed high blood pressure (175/75 mmHg) without ocular bruit or audible murmur on auscultation. Optical coherence tomography of the macula showed inner retinal thickening over the temporal macula. Ultrasound carotid Doppler and computed tomography angiography of the carotid showed more than 75% stenosis over the right distal internal carotid artery.

Unfortunately, she developed rubeosis iridis over her right eye two weeks after her presentation, which required pan-retinal photocoagulation. She subsequently progressed to NVG, requiring maximum anti-glaucoma medications to stabilize intraocular pressure. In conclusion, CRAO is a sight-threatening medical emergency. Thorough investigations are required to determine the underlying cause so that early intervention can be done to reduce the risk of a similar attack in the fellow eye and the risk of a cerebrovascular event or cardiac ischemia, which could be life-threatening. The presence of a cilioretinal artery does not prevent ocular neovascularization in CRAO. Hence, patients should also be closely monitored after the initial diagnosis to prevent devastating complications such as NVG.

## Introduction

Central retinal artery occlusion (CRAO) is a sight-threatening condition and is regarded as an ocular emergency. Literally, it is an ischaemic type of stroke, and patients have an associated risk of developing subsequent vascular events in the future [1]. Patients with CRAO typically present with acute, profound, painless unilateral blurring of vision. Most of the affected individuals have a final visual acuity of counting fingers or worse. Like Madike's study, Mac GB et al. also reported that only less than 20% of the cases have regained functional visual acuity in the affected eye [2, 3]. A distinct variant of CRAO with the presence of a cilioretinal artery has a more favorable outcome, as the visual acuity can recover to 20/50 or better in over 80% of eyes. However, the cilioretinal artery is only present in about one-third of the eyes [4]. The aim of this case report is to discuss a case of unilateral CRAO with cilioretinal artery sparing, which was thought to be a good prognostic factor for the patient who developed neovascular glaucoma within two weeks of diagnosis.

## Case presentation

A 75-year-old Indian lady with underlying osteoarthritis and bilateral chronic otitis media was under ophthalmology clinic follow-up for bilateral pseudophakia and normal tension glaucoma (NTG), which was treated with two topical anti-glaucoma drops (gutt timolol 0.5% twice daily (BD) and gutt Xalatan once every night (ON)). Her baseline best corrected visual acuity (BCVA) was 6/9 bilaterally. She presented with sudden onset right eye painless loss of vision. The loss of vision was generalized and was noticed upon waking up from sleep. She denied any eye redness or discharges, flashes of light, floaters, or any recent ocular trauma prior to the loss of vision. There was no previous episode of amaurosis fugax. She also did not have any fever, jaw claudication, scalp pain, or headache suggesting giant cell arteritis (GCA). She presented to our clinic one week after the onset due to no visual recovery.

**Figure 1:**
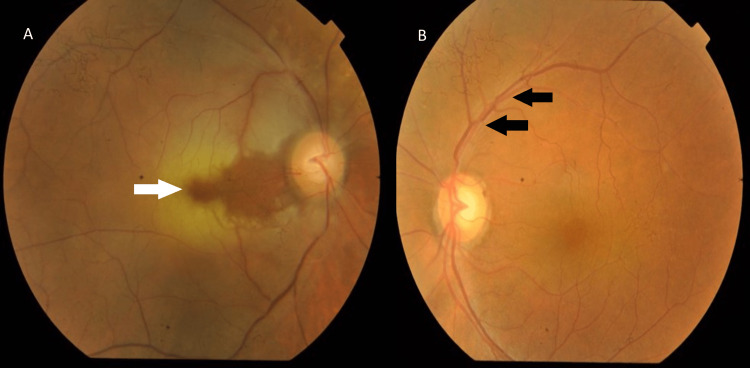
A colored fundus photograph shows (A) right eye generalized retinal arteriolar attenuation and macular whitening sparing the papillomacular bundle (white arrow) with glaucomatous optic disc changes; (B) left eye grade II hypertensive retinopathy evidenced by copper wiring and arteriovenous nicking over the superotemporal arcade (black arrow) with glaucomatous optic disc changes.

Upon presentation to the eye casualty, her visual acuity (VA) was hand motion (HM) over the right eye and 6/12 over the left eye, with the presence of a right eye relative afferent pupillary defect (RAPD). The anterior segment findings for both eyes were unremarkable. The intraocular pressure (IOP) was 14 mmHg in both eyes. The right eye fundus revealed retinal whitening at the macular region sparing the papillomacular bundle and generalized retinal arteriolar attenuation, as shown in Figure 1A.

Otherwise, the optic disc was still pink with a cup-to-disc ratio (CDR) of 0.7. There was no new vessel seen at the disc or elsewhere. The left eye fundus showed hypertensive retinopathy changes and similar glaucomatous optic disc changes in the right eye, with a CDR of 0.8 as shown in Figure 1B. Systemic examination showed a raised blood pressure of 175/75 mmHg with a normal and regular pulse rate. There was no scalp tenderness, carotid bruit, or audible murmur on the cardiovascular examination.

**Figure 2:**
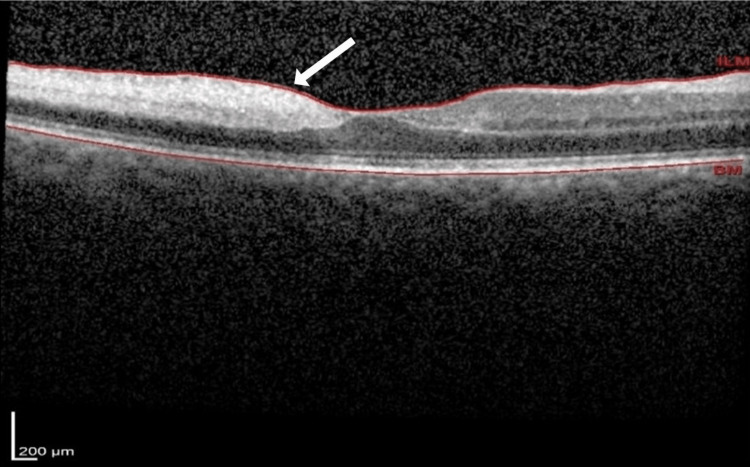
Optical coherence tomography of the right eye macula shows thickening and hyper-reflectivity of the inner retinal layer temporal to the fovea (white arrow) with normal retinal configuration over the nasal macula.

No intervention was carried out to restore the retinal blood flow due to the late presentation. However, she was investigated for the cause of CRAO. Her blood investigations showed a normal full blood count, fasting serum lipids, and blood sugar with a hemoglobin A1C (HbA1C) of 5.8%. Her erythrocyte sedimentation rate (ESR) was 26 mm/hr, which was normal for her age. Optical coherence tomography (OCT) of the right eye macula showed inner retinal thickening, which was confined to the temporal macula, as shown in Figure 2.

Her ultrasound carotid Doppler showed less than 50% stenosis over the right distal common carotid artery (CCA) and proximal internal carotid artery (ICA), with similar findings over the left side. Echocardiography (ECHO) showed an ejection fraction of 68%, with no valve abnormalities and no regional wall motion abnormality.

**Figure 3:**
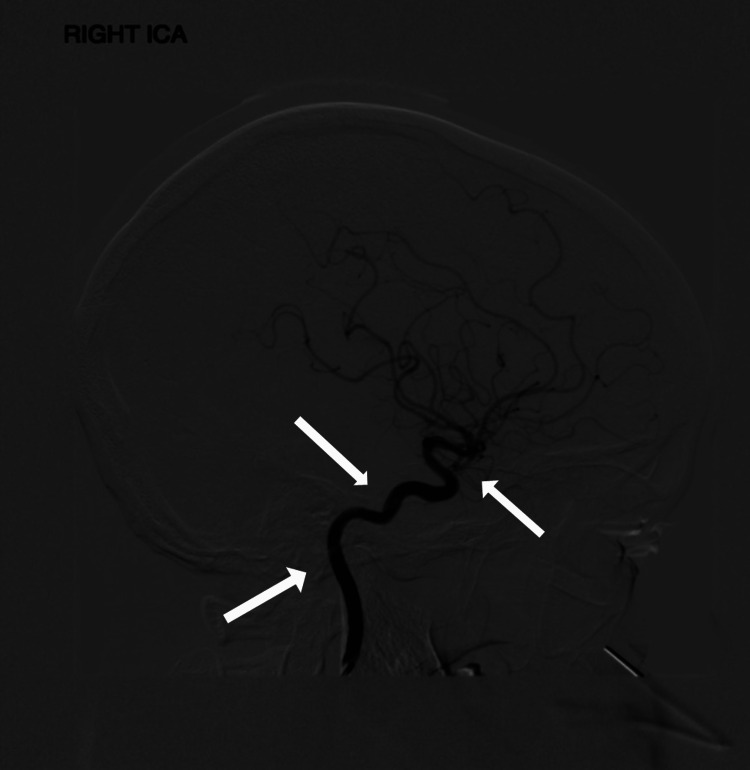
The carotid angiogram shows areas of progressive narrowing of the right internal carotid artery.

Subsequently, a computed tomography angiography (CTA) of the brain, circle of Willis, and carotid arteries was performed and showed atherosclerotic diseases of bilateral extracranial ICA and significant stenosis affecting the right proximal ICA with approximately 75% stenosis. The carotid angiogram also showed progressive narrowing of the right ICA, with diameters at the proximal, mid, and distal regions measuring 9.6mm, 5.8mm, and 5.5 mm, respectively (Figure 3).

The patient was managed both by the neuromedical and neurosurgical teams for her right ICA stenosis. She was started on anti-hypertensive, anti-platelet, and statins to stabilize the blood pressure and prevent another ischaemic event that could potentially be life-threatening or sight-threatening for the contralateral eye. She then underwent a right carotid endarterectomy performed by the neurosurgical team, which was uneventful.

**Figure 4:**
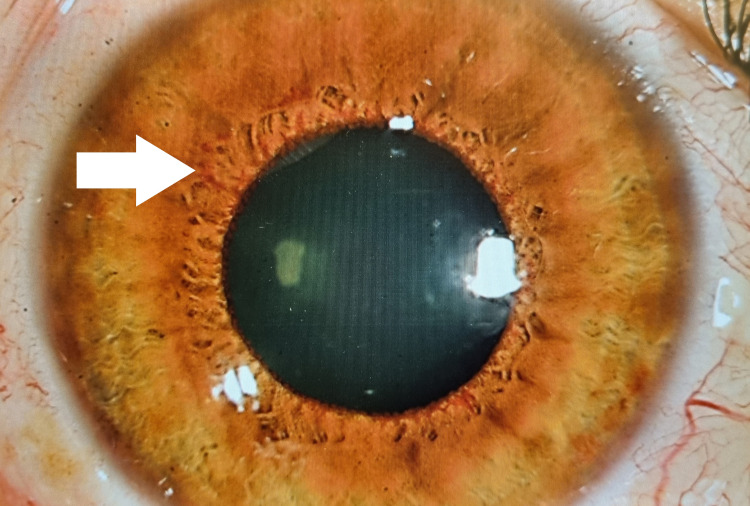
Rubeosis iridis is seen at the pupillary margin area, almost 360 degrees.

Unfortunately, this patient developed right eye rubeosis iridis with new vessels formed over the pupillary margin and anterior chamber angle two weeks after her presentation, as shown in Figure 4.

**Figure 5:**
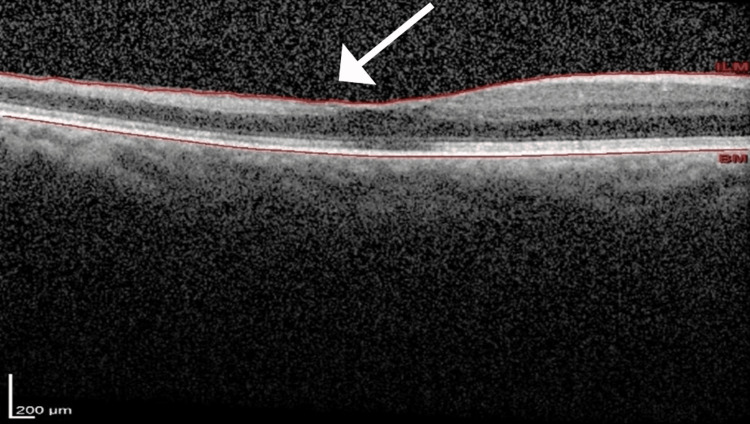
Optical coherence tomography of the right eye macula shows thinning of the inner retinal layer temporal to the fovea in comparison to the nasal region.

Full pan-retinal photocoagulation was commenced for her right eye in multiple sessions. She subsequently developed neovascular glaucoma (NVG) with an increasing IOP trend up to 38 mmHg. She was started on another two topical anti-glaucoma products (gutt Azopt BD and gutt Alphagan BD) on top of her initial two eyedrops. Nevertheless, her right eye vision remained as hand motion, with a pale optic disc and CDR of 0.9. The repeated OCT macula of the right eye showed inner retinal layer atrophy at the temporal macula, as shown in Figure 5.

## Discussion

Central retinal artery occlusion (CRAO) is caused by sudden occlusion of the central retinal artery (CRA). Anatomically, CRA is the first branch of the ophthalmic artery (OA), which is then divided into superior and inferior branches and subsequently further divided into nasal and temporal branches to supply the inner two-thirds of the retina layer. The outer one-third of the retina is supplied by the choriocapillaris, which originates from the short posterior ciliary artery (SPCA) [2, 5]. Generally, only 20% of the population has extra arterial supplies to the macula from the cilioretinal arteries of the SPCA [6]. Justice J. et al. showed 49.5% of patients in 1,000 subjects have a patent cilioretinal artery based on fundus fluorescein angiography (FFA) findings [7]. Meanwhile, Jackson E. reported that cilioretinal arteries were present in 191 of 500 eyes, with 43 of them having two cilioretinal arteries. Of the 43 eyes, 12 had double cilioretinal artery circulation from independent origins [8].

Central retinal artery occlusion can be classified based on its etiology. There are four distinct clinical entities, which include non-arteritic CRAO, non-arteritic transient CRAO, non-arteritic CRAO with cilioretinal artery sparing, and arteritic CRAO with GCA [3-4, 9]. Kim et al. reported that 17.8% of their CRAO cases showed cilioretinal artery sparing [6]. There are several risk factors associated with CRAO, which include hypertension, diabetes mellitus, carotid artery disease, coronary artery disease, and stroke [2-3, 6]. Atherosclerosis carotid artery disease also shares similar risk factors, including diabetes mellitus, hypertension, male gender, smoking, drinking, and advanced age [10]. Regardless of age, postmenopausal women are at higher risk of developing atherosclerotic disease and cardiovascular disease due to the loss of the protective effect of endogenous estrogen compared to age-matched males [11]. The most common cause of non-arteritic CRAO is strongly associated with ipsilateral ICA stenosis [12]. Our patient was a postmenopausal woman with undiagnosed hypertension that predisposed her to stenosis of bilateral ICA and subsequently led to this devastating ocular complication.

In this case report, the profound loss of vision at presentation and the appearance of generalized whitening of the retina sparing the papillomacular bundle were very suggestive of CRAO with cilioretinal artery sparing. The papillomacular bundle is a collection of nerve fibers that carries information from the macula to the optic nerve, which is important for central vision. Cases of CRAO with cilioretinal artery sparing have a better prognosis for their dual supply, which is mainly from the CRA and additional blood supply from choriocapillaries. Theoretically, it protects the macula from infarction in the event of CRAO. Hence, in the presence of a cilioretinal artery, visual acuity usually recovers to 20/50 or better in over 80% of eyes [13]. On the contrary, Kim et al. showed 81.3% of the CRAO cases with cilioretinal artery sparing had final visual acuity worse than 20/200, but the incidence was still lower than non-sparing CRAO, which was 97.3% [6]. Despite the presence of a cilioretinal artery in our patient, her visual acuity was hand motion, most likely due to late presentation causing irreversible retinal damage, and possibly only a small area of the papillomacular bundle was being perfused by her cilioretinal artery.

In some cases, the cilioretinal artery can be visualized by dilated fundus examination and fundus photography. To confirm the patency of the cilioretinal artery, FFA should ideally be performed, however in this case it was deferred at the initial presentation. In CRAO, FFA may reveal central retinal artery filling delay and arterio-venous transit time delay [6, 14-15]. In cases where FFA cannot be done due to allergies or kidney disease, optical coherence tomography angiography (OCT-A) can be performed to look at the blood flow of the superficial and deep capillary retinal plexus [14, 15]. In CRAO with cilioretinal artery sparing, OCT-A may show the normal flow of the macular area, but disruption of the flow signs in the superficial retinal layer, deep plexus, and choriocapillaris is due to edema of the inner layer of the retina [14, 15]. Optical coherence tomography angiography is not commonly performed due to difficult fixation, long acquisition time, and motion artifacts [15]. Ultrasound carotid Doppler and carotid angiograms help to confirm the carotid artery stenosis, which was performed in our case.

The classical symptom of CRAO is a sudden, unilateral, painless loss of vision of various degrees. Similarly, our patient presented with sudden, generalized loss of vision without any improvement after a week. Her ultrasound carotid Doppler and CTA of carotid arteries showed evidence of narrowing and stenosis of bilateral ICA, which is suggestive of atherosclerotic disease. The occlusion of the CRA is commonly caused by emboli, which results in retinal ischemia. Central retinal artery occlusion can lead to undetectable optic nerve or retinal damage if the occlusion lasts for less than 90 minutes, whereas after 240 minutes, retinal infarction occurs [5-6, 9]. When retinal ischemia occurs, there will be retinal thickening, in which the hyperreflectivity of the inner retinal layer can be visualized on the OCT. A prolonged period of ischemia lasting more than 240 minutes can lead to retinal infarction, which explains the poor visual recovery in our patient. As a consequence, the viable retinal cells will produce vascular endothelial growth factor (VEGF) and lead to ocular neovascularization [16, 17].

In acute CRAO presentation, there are few immediate treatments that can be offered, ideally within four hours from the onset of loss of vision such as (1) ocular massage (cycle of applying ocular pressure with Goldmann 3-mirror for 10 seconds followed by five seconds of rest and then look at the centre mirror whether visible emboli has been dislodged distally), (2) anterior chamber paracentesis with a 27G or 30G needle by withdrawing 0.1-0.4 ml of aqueous, (3) oral or intravenous acetazolamide with topical anti-glaucoma, or (4) re-breathing in a bag with a carbogen therapy (95% oxygen and 5% carbon dioxide) [3, 5]. These techniques can be performed as they help either to lower the intraocular pressure or to vasodilate with the aim of dislodging the emboli distally and reducing the area of retinal ischemia [3, 5]. Referral to the emergency department or neuromedical team for a trial of thrombolysis therapy, as this is analogous to ischaemic stroke [3]. Nevertheless, there are no evidence-based therapies that have demonstrated efficacy in improving visual outcomes in CRAO. To prevent recurrent or secondary attacks, blood investigations such as full blood count, erythrocyte sediment rate, and c-reactive protein can aid in diagnosing GCA. Pan-retinal photocoagulation helps reduce the retinal oxygen demand and reduces the risk of NVG. Anticoagulant should be started if there is no contraindication, as it reduces the risk of thromboembolic in atherosclerotic disease [3]. Similarly, in our case, the patient was started on antiplatelet and statins to prevent recurrent attacks for her ICA stenosis and ultimately right carotid endarterectomy to improve the arterial blood flow.

Despite having a patent cilioretinal artery, our patient developed rubeosis iridis, which eventually led to NVG requiring additional topical anti-glaucoma medication to control the IOP. Rudkin et al. showed a prevalence of neovascularization in their study of 18.2% at an average mean of 8.5 weeks following the onset of CRAO (range: two to 16 weeks) [18]. Retinal neovascularization is uncommon. Ducker et al. reported that about 1.8% (three out of 168) of patients with CRAO developed neovascularization of the disc (NVD), with two of them having rubeosis iridis [19]. Meanwhile, Jung YH et al. reported that the incidence of rubeosis iridis was 10.9% with a mean time of three months following acute CRAO [20]. None of the studies reported the prevalence of ocular neovascularization, specifically in CRAO with cilioretinal artery sparing. Interestingly, our patient developed neovascularization at the iris and angle at a very early stage, which was two weeks after her presentation.

## Conclusions

Even though CRAO is a sight-threatening condition with poor visual prognosis, thorough investigations are required to determine the underlying cause so that early intervention can be done to reduce the risk of a similar attack in the fellow eye and the risk of a cerebrovascular event or cardiac ischemia, which could be life-threatening. The presence of a cilioretinal artery does not prevent ocular neovascularization in CRAO. Hence, patients should also be closely monitored after the initial diagnosis to prevent devastating complications such as NVG.
